# The Involvement of HLA Class II Alleles in Multiple Sclerosis: A Systematic Review with Meta-analysis

**DOI:** 10.1155/2019/1409069

**Published:** 2019-11-06

**Authors:** A. De Silvestri, C. Capittini, G. Mallucci, R. Bergamaschi, C. Rebuffi, A. Pasi, M. Martinetti, C. Tinelli

**Affiliations:** ^1^Clinical Epidemiology and Biometric Unit, IRCCS Policlinico S. Matteo Foundation, Viale Golgi 19, 27100 Pavia, Italy; ^2^Department of Biology and Biotechnology “Lazzaro Spallanzani”, University of Pavia, Italy; ^3^Inter-Department Multiple Sclerosis Research Centre, IRCCS Mondino Foundation, Pavia, Italy; ^4^Scientific Documentation Service, IRCCS Policlinico S. Matteo Foundation, Viale Golgi 19, 27100 Pavia, Italy; ^5^Department of Transfusion Medicine and Immuno-Hematology (Laboratory of Immunogenetics), IRCCS Policlinico S. Matteo Foundation, Viale Golgi 19, 27100 Pavia, Italy

## Abstract

Multiple Sclerosis (MS) displays a heterogeneous clinical onset and progression, which are mostly unpredictable, but demyelination of the central nervous system (CNS) leads to substantial deficits of sensory, motor, autonomic, and neurocognitive functions. Considering all genetic studies on MS, including the advanced genome-wide association studies, the risk linked to HLA alleles remains the highest among other susceptibility genetic variants. However, given the genetic variability of HLA alleles in different ethnic groups, we conducted a systematic review of reviews and meta-analyses aiming at summarizing all the results on the association between MS and HLA class II genes. We systematically searched meta-analyses and systematic reviews dealing with MS and HLA in all ethnicities. From 154 records, we included 5 articles collecting HLA data from 15,232 MS patients and 24,194 ethnically matched controls. DRB1∗15 (OR ranging from 1.39 in Chinese Han to 2.59 in Caucasians) and DQB1∗06:02 (OR ranging from 1.91 in Caucasians to 2.49 in Colombian) alleles confer an increased risk for MS transethnically (Caucasians, Chinese, South Americans, Carribeans, Middle Easterners, Japanese, and North Africans). DRB1∗01, DRB1∗09, DRB1∗11, DRB1∗12, and DRB1∗16 alleles were protective, in agreement with the type of amino-acidic (aa) residues (ranging from position 9 to 90) included in pockets 1, 4, 6, 7, and 9, which are most involved in peptide presentation. Changes in aa residues affect the capability of HLA molecules in binding myelin peptides. DQB1∗06:02 risk allele seems to be the most interesting target as humanized mice expressing only DQB1∗06:02 develop MS-like disease mediated by autoimmune reactions against myelin oligodendrocytic basic protein that stabilizes the myelin. Our summary of results from a high number of patients and controls suggests that allelic variants from both DQB1 and DRB1 genes are equally involved in MS susceptibility/protection transethnically.

## 1. Introduction

Multiple Sclerosis (MS) is an inflammatory demyelinating disease of the central nervous system (CNS) that can lead to substantial deficits of sensory, motor, autonomic, and neurocognitive functions.

The disease is known to display a heterogeneous clinical onset and progression, which are mostly unpredictable [1]. According to the *Atlas of MS*, it approximately affects 2,3 million people worldwide and typically presents between the ages of 20 and 40. Women are 2 to 3 times more susceptible to MS compared to men [2].

Current data support the view of MS as a multifaceted disease caused by complex interactions between environmental factors and genetic susceptibility [3]. On one hand, ethnicity, geographical location, and environmental factors (such as vitamin D, smoke, infections, and air pollutions) influence both incidence and prevalence; on the other hand, due to an increased frequency of MS among first-degree relatives of affected subjects, a genetic background has been invoked to explain family aggregation [4].

Epidemiological studies have shown that genetic factors are primarily responsible for SM predisposition, and linkage studies in multiplex families have confirmed that variation within the Human Leukocyte Antigen (HLA) region exerts the greatest individual effect on MS risk [5]. Over the past 10 years, genome-wide association studies (GWAS) have identified more than 200 risk variants independently contributing to MS susceptibility, but none of them showed to be a risk factor as high as HLA alleles, as the strongest genetic association signal still resides in chromosome 6p21.3, the HLA region [6].

The most recent GWAS conducted by the International Multiple Sclerosis Genetics Consortium (IMSGC) analyzed genetic data from 47,351 MS patients and 68,284 healthy subjects to build a complex genetic architecture of MS, including 200 autosomal variants, one chromosome X variant, and 32 independent variants in the HLA extended region. The 201 non-HLA variants accounted for 19.2% of the total MS heritability, while 32 HLA associations account for 4%. While the autosomal dissection with high-throughput sequencing is feasible, the HLA region displays multiple alleles leading to complex haplotypes in different populations. For this reason, the IMSGC published a comprehensive dissection of allelic association in the extended HLA region with dense genotyping on 48,000 samples [7].

Here, we provide an appraisal focused on the role of HLA-DRB1 and HLA-DQB1 polymorphisms in MS revising data from meta-analyses and systematic reviews on this topic and giving the clinicians a final easy to handle synoptic table for all major ethnicities.

## 2. Materials and Methods

### 2.1. Protocol

We drew up a protocol including review questions, selection and eligibility criteria, primary outcome, search strategy, methods for data extraction, methods for assessing study quality, and risk of bias. On 21 December 2017, the protocol entitled “Association between HLA Class II (DRB1) Polymorphisms and Multiple Sclerosis: A Review of Systematic Reviews” was published in the PROSPERO International prospective register of systematic reviews (PROSPERO CRD42017076831), and from that date forward, it is available from https://www.crd.york.ac.uk/PROSPERO/display_record.php?RecordID=76831.

The type of our review was “review of reviews.” The review aim was “To review the association between Multiple Sclerosis and Human Leukocyte Antigen class II polymorphisms.” Types of studies included were published meta-analysis and systematic reviews that involved case-control studies on genetic association between HLA-DRB1 and DQB1 and MS. Reviews and single research articles (not included yet in meta-analysis or systematic review) were excluded. No exclusion was made based on ethnicity.

Participants were patients with MS diagnosed following these clinical and diagnostic criteria:

1. Criteria from 2001 to 2004: McDonald criteria [8]
2. Criteria from 2005 to 2010: Revised McDonald criteria [9]
3. Criteria from 2011: 2010 Revised McDonald criteria [10]

The primary outcome was OR measuring the association between HLA-DRB1 and MS presented in a “summary of evidence” table.

### 2.2. Search Strategy

We performed a systematic search of PubMed, EMBASE, Web of Science, Cochrane databases, and Scopus retrieving all publications (meta-analysis and systematic reviews of case-control studies) on the association between HLA class II (DRB1) polymorphisms and MS in adults (>18 years). We searched all English articles published from inception to September 2019.

The search strategy in PubMed was “Multiple Sclerosis” (Mesh) AND “HLA-DR Antigens” (Mesh). The search strategy in Web of Science was TS = “Multiple Sclerosis” AND HLA DRB1. The search strategy in EMBASE was “Multiple Sclerosis”/exp AND “hla drb1 antigen”/exp. The search strategy in Scopus was multiple sclerosis (index term) and HLA DRB1 (index term). The search strategy in Cochrane was “Multiple Sclerosis” (Mesh). Selection criteria were HLA class II (DRB1) polymorphisms and Multiple Sclerosis diagnosed following the clinical criteria from Poser et al. [8–11].

Two reviewers working independently and with adequate reliability ascertained the eligibility of each study included in our review by evaluating 11 items from the AMSTAR checklist [12].

### 2.3. Data Extraction and Analysis

**Figure 1:**
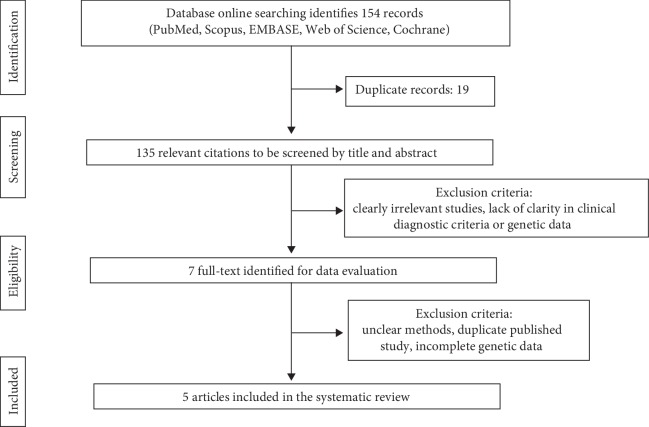
Flow diagram of the study following the PRISMA statement.

After a critical reading of the articles, two investigators independently performed data extraction according to the inclusion criteria listed above. The third participant was consulted for discussion to reach agreement concerning discrepancies. The following items were extracted from each study: first author's last name, publication date, country of origin, numbers of studies included, total number of cases and controls, typing method, statistical analysis, and Odds Ratio (OR).  Figure 1 shows the complete flow diagram following the PRISMA statement [13].

Stata 14.2 (StataCorp, USA) was used for statistical analysis to perform the meta-analysis on data from Lima et al. Heterogeneity was checked by the Chi-squared test and the *I*-squared statistic. The criteria for identification of heterogeneity were the *p* value less than 0.10 for the Chi-squared test and an *I*-squared statistic greater than 50%. When there was no statistical evidence for heterogeneity in effect sizes, we used the fixed effects model to meta-analyze the Odds Ratio (OR) in probands; when significant heterogeneity was identified, we used the random effects model.

### 2.4. Strategy for Data Synthesis

We listed all the HLA class II alleles found associated with MS in our search in a clear and simple “summary of evidence” table of results (visual “stop-light” indicator, where light grey indicates the beneficial or protective alleles, dark grey indicates the detrimental or disease-predisposing alleles, and white indicates neutral alleles that is neither protective nor detrimental).

## 3. Results

### 3.1. Study Selection and Characteristics

Our search strategy yielded 154 records for consideration (Figure 1). Following elimination of the 19 duplicates, 135 titles were reviewed.

Of these, 128 were excluded: of the remaining 7 publications, full-text manuscripts were obtained and assessed for eligibility, and 2 articles were excluded. Finally, 5 publications were deemed eligible for inclusion and were submitted to data extraction: 4 meta-analyses [14–17] and 1 systematic review [18]. Articles were published between 2010 and 2018. All articles included patients with a diagnosis of MS. Therefore, as shown in Table 1, 15,232 MS patients and 24,194 ethnically matched controls were included.

**Figure 2:**
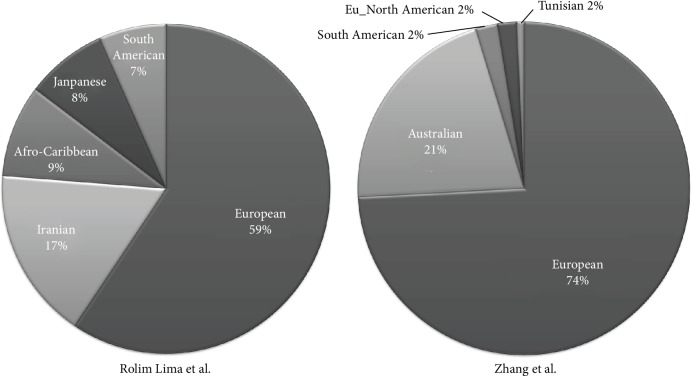
Pie charts representing percentages of ethnicities included in Rolim Lima et al. and Zhang et al. articles [16, 18].

Overall, we included different ethnicities: Chinese Han from Qiu et al., Colombian from Rojas et al., Middle Easterner and North Africans from Mohajer et al., 97% Caucasians from Zhang et al. (2% South American, 1% North Africa), 59% Caucasians from Rolim Lima et al. (17% Middle Eastern, 9% Carribean, 7% South American, and 7% Japanese) (Figure 2) [14–18].

### 3.2. Quality of Studies

The quality of studies was evaluated following 11 items of the AMSTAR checklist. All studies showed an a priori design; nevertheless, none of them considered the grey literature and assessed the scientific quality of the included studies (Supplementary data [Supplementary-material supplementary-material-1]).

### 3.3. Meta-analysis on the Association between MS and HLA Polymorphisms from Rolim Lima et al.

As we included in our systematic review 4 meta-analyses [14–17] and 1 systematic review [18], we extracted HLA data from the only one systematic review and meta-analyzed them.

**Figure 3:**
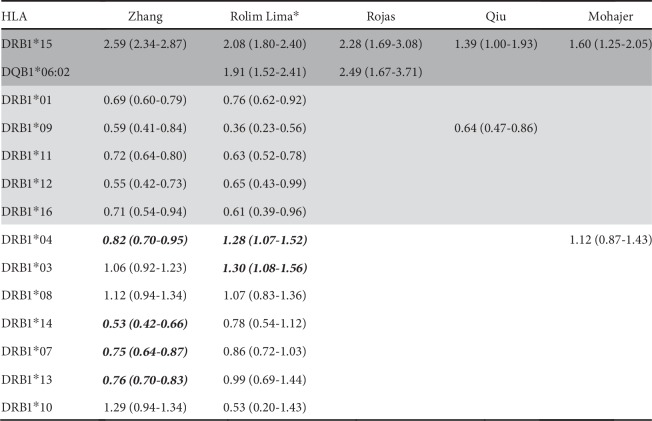
Association between MS susceptibility/protection and HLA-DRB1 alleles and HLA-DQB1∗06:02. Dark grey Odds Ratio (OR) indicates the disease-predisposing alleles, and light grey OR indicates the protective alleles. White bold OR indicates those alleles that are protective in some ethnicities or predisposing in other ones. White OR indicates nonsignificant results.

We performed a meta-analysis on the case-control studies included in the systematic review from Rolim Lima and colleagues [18] and reported the corresponding OR for HLA alleles in Figure 3. We found that HLADRB1∗15, HLA-DQB1∗06:02, and HLA-DRB1∗03 alleles conferred an increased risk for MS; HLADRB1∗01, HLA-DRB1∗09, HLA-DRB1∗11, HLA-DRB1∗12, and HLA-DRB1∗16 alleles resulted protective for MS; all other alleles listed in Figure 3 were nonstatistically significant (Supplementary data [Supplementary-material supplementary-material-1]).

### 3.4. Summary on the Association between MS Susceptibility and HLA Polymorphisms

Overall, HLA-DRB1∗15 and HLA-DQB1∗06:02 alleles confer an increased risk for MS. On the contrary, HLA-DRB1∗01, HLA-DRB1∗09, HLA-DRB1∗11, HLA-DRB1∗12, and HLA-DRB1∗16 alleles are protective (Figure 3).

As to other alleles, we have conflicting results: HLA-DRB1∗03 is predisposing in the ethnically mixed group [18], while HLA-DRB1∗07, HLA-DRB1∗13, and HLA-DRB1∗14 are protective in the group with the highest percentage of Caucasians [16] (Figure 3).

HLA-DRB1∗04 is protective in the group with the highest percentage of Caucasians [16] and predisposing in the ethnically mixed group [18] and also predisposing in Middle Easterners and North Africans [17] although without statistical significance (Figure 3).

HLA-DRB1∗08 and HLA-DRB1∗10 show opposite results without statistical significance in any group (Figure 3).

## 4. Discussion

This systematic review is focused on the association between Human Leukocyte Antigen (HLA) class II polymorphisms and susceptibility or resistance to Multiple Sclerosis (MS). We unveil some limitations of this revision as the included meta-analyses and systematic reviews evaluating the HLA-related genetic background in MS patients showed low quality according to the AMSTAR checklist (Supplementary data [Supplementary-material supplementary-material-1]): no quality assessment was present (PRISMA statement), and the grey literature was not included [12, 13]. On the contrary, all studies showed an a priori design, provided the characteristics of included studies, and used an appropriate method to combine the findings. This last observation allows us to be confident with the immunogenetic data that we analyzed.

We know that HLA alleles are under positive natural selection in different ethnical groups due to the pivotal role of peptide presenters to lymphocytes, and this could be a reason why some alleles may display opposite effects (susceptibility/protection to pathologies) in different ethnicities. However, reviewing genetic data from 15,232 MS patients and 24,194 ethnically matched controls (Table 1), we confirm the pivotal role of HLA-DRB1∗15 as the highest MS risk factor in the HLA region across several ethnicities: Caucasians, Han Chinese, Colombians, Middle Easterners, Caribbean, South Americans, Japanese, South Americans, and North Africans (Figure 2).

Besides HLA-DRB1∗15, we found an association between MS risk and HLA-DQB1∗06:02 in Colombians [15] and in the mixed group composed of Caucasians, Middle Eastern, Japanese, Carribean, and South American [18] (Figure 3). This seems not surprising if we consider the linkage frequently observed between these HLA variants in different populations, above all in Caucasians. However, further insights have emerged by studies on murine models. Kaushansky and Ben-Nun observed a susceptibility of DRB1∗15:01 transgenic mice to develop MS-like disease induced by autoimmune reactions against myelin basic protein or myelin oligodendrocyte glycoprotein, but they also observed a susceptibility of humanized mice expressing only HLA-DQB1∗06:02 (not DRB1∗15:01) to develop MS-like disease mediated by autoimmune reactions against myelin oligodendrocytic basic protein, a central nervous system (CNS) myelin-specific protein that stabilizes the myelin [19]. Different molecular targets deriving from the CNS peptidome may be responsible of modulating the phenotype and inducing different responses to therapies. For this reason, the cases of MS patients carrying HLA-DRB1∗15 without HLA-DQB1∗06:02 (or vice versa) are important to settle the role of each gene and allele in MS susceptibility. In particular, researchers identified Norwegian MS patients who carried HLA-DQB1∗06:02 without HLA-DRB1∗15, but carriers of HLA-DRB1∗15 always showed also HLA-DQB1∗06:02. In Afro-Brazilians, HLA-DQB1∗06:02 showed a higher association than HLADRB1∗15, while Afro-American HLA-DRB1∗15 showed a higher association than HLA-DQB1∗06:02. Besides HLA allelic analysis, we studied the amino-acidic (aa) polymorphisms in those HLA-DRB1 molecules protective for MS. We considered those 19 aa residues (ranging from position 9 to 90) that are included in pockets 1, 4, 6, 7, and 9 which are most involved in peptide presentation [5]. Considering the HLA-DRB1 protein encoded by the HLA-DRB1∗15:01 risk allele as reference, we analyzed the changes in all 19 aa residues from those proteins encoded by 7 HLA-DRB1 alleles found to be protective for MS (Figure 3).

We found a gradient of amino-acidic differences going from the less different to the most different proteins, and here, we list the corresponding alleles: HLA-DRB1∗09:01 codes for a molecule with 10/19 different aa variants; HLA-DRB1∗01:01/02 with 6/19 aa variants; HLA-DRB1∗12:01 with 5/19 aa variants; HLADRB1∗11:01 AND HLA-DRB1∗16:01 with 1/19 aa variants; and HLA-DRB1∗11:04 with 0/19 aa variants. Noteworthily, HLA-DRB1:11∗04 seems nonprotective as it shares the same aa compared to HLADRB1∗15:01 alleles.

As the most characterizing HLA-DRB1 alleles for MS protection are HLA-DRB1∗09 and HLA-DRB1∗01 (with 10 and 6 aa changes, respectively), accordingly, pockets characterizing protection against MS are 1, 4, and 6.

In pocket 1, at position 86, many alleles show Glycine (G) instead of Valine (V). Although these two aa are nonpolar and hydrophobic, this dimorphism affects the stability of the HLA-DRB1 molecule on the surface and thus peptide presentation. In particular, G86 confers surface instability to the molecule, with the consequence of presenting myelin peptides to CD4 T lymphocytes for a shorter time, thus possibly reducing the immune response [20].

DRB1∗09:01 allele, unique among all other protective alleles, shows a glutamic acid (E) at position 74, thus giving a negative charge to pocket 4. All other alleles show positively charged aa or nonpolar and hydrophobic like alanine or leucine.

Pocket 4 polarity seems to be important in binding the myelin basic protein (MBP), as it has been shown that the immune response is directed against MBP in MS patients; thus, E74 might prevent MBP binding conferring protection [21]. Moreover, a negative-charged aa at position 74 seems to be protective in the course of patients affected by the relapsing-remitting MS [22].

HLA-DRB1∗09 and HLA-DRB1∗01 alleles share phenylalanine (F) at position 13 in pocket 4. F shows a bulky aromatic ring in the side chain that can reduce the number of peptides, including myelin, and thus conferring protection to MS. On the contrary, a positively charged or a neutral aa at position 13 confers susceptibility to several autoimmune disease, first of all rheumatoid arthritis [23].

Finally, HLA-DRB1∗04 is significantly predisposing in the ethnically mixed group (Rolim Lima et al.) and significantly protective in the group with the highest percentage of Caucasians (Zhang et al.) (Figure 3). HLADRB1∗14, HLA-DRB1∗07, and HLA-DRB1∗13 are significantly protective in the group with the highest percentage of Caucasians (Zhang et al.) and protective in the ethnically mixed group but without statistical significance (Rolim Lima et al.) (Figure 3). HLA-DRB1∗03 is significantly predisposing in the ethnically mixed group (Rolim Lima et al.) and predisposing in the group with the highest percentage of Caucasians (Zhang et al.) but without statistical significance. HLA-DRB1∗08 is predisposing in both groups but without statistical significance. Thus, some HLA alleles seem to be influenced by ethnical ancestry in predisposing or protecting from MS.

As our summary of results from a high number of patients and controls suggests that allelic variants from both DQB1 and DRB1 are equally involved in MS susceptibility/protection transethnically, it seems to be useful to type both DRB1 and HLA-DQ alleles in MS patients.

## Figures and Tables

**Table 1:** 15,232 MS patients and 24,194 ethnically matched controls included in this review.

Article	Included studies	Patients	Controls
Qiu et al. [14]	6	308	407
Rojas et al. [15]	7	464	2581
Zhang et al. [16]	32	10865	15347
Rolim Lima et al. [18]	9	1797	2945
Mohajer et al. [17]	10	1798	2914

## Supplementary Materials

Supplementary Materials Supplementary data Figure 1: analysis of the quality of the studies included in the present review of systematic reviews. Supplementary data Figures 2-10: forest plots from the meta-analysis including HLA data from Rolim Lima et al.

## References

[1] Compston A., Coles A. (2002). Multiple sclerosis. *The Lancet*.

[2] Browne P., Chandraratna D., Angood C. (2014). Atlas of Multiple Sclerosis 2013: a growing global problem with widespread inequity. *Neurology*.

[3] Dobson R., Giovannoni G. (2019). Multiple sclerosis – a review. *European Journal of Neurology*.

[4] Bergamaschi R., Cortese A., Pichiecchio A. (2018). Air pollution is associated to the multiple sclerosis inflammatory activity as measured by brain MRI. *Multiple Sclerosis*.

[5] Hollenbach J. A., Oksenberg J. R. (2015). The immunogenetics of multiple sclerosis: a comprehensive review. *Journal of Autoimmunity*.

[6] International Multiple Sclerosis Genetics Consortium (2011). Genetic risk and a primary role for cell-mediated immune mechanisms in multiple sclerosis. *Nature*.

[7] International Multiple Sclerosis Genetics Consortium, Patsopoulos N. A., Baranzini S. E. (2019). The multiple sclerosis genomic map: role of peripheral immune cells and resident microglia in susceptibility. *bioRxiv*.

[8] McDonald W. I., Compston A., Edan G. (2001). Recommended diagnostic criteria for multiple sclerosis: guidelines from the international panel on the diagnosis of multiple sclerosis. *Annals of Neurology*.

[9] Polman C. H., Reingold S. C., Edan G. (2005). Diagnostic criteria for multiple sclerosis: 2005 revisions to the “McDonald Criteria”. *Annals of Neurology*.

[10] Polman C. H., Reingold S. C., Banwell B. (2011). Diagnostic criteria for multiple sclerosis: 2010 revisions to the McDonald criteria. *Annals of Neurology*.

[11] Poser C. M., Paty D. W., Scheinberg L. (1983). New diagnostic criteria for multiple sclerosis: guidelines for research protocols. *Annals of Neurology*.

[12] Shea B. J., Hamel C., Wells G. A. (2009). AMSTAR is a reliable and valid measurement tool to assess the methodological quality of systematic reviews. *Journal of Clinical Epidemiology*.

[13] Moher D., Liberati A., Tetzlaff J., Altman D. G., PRISMA Group (2009). Preferred reporting items for systematic reviews and meta-analyses: the PRISMA statement. *BMJ*.

[14] Qiu W., James I., Carroll W. M., Mastaglia F. L., Kermode A. G. (2011). HLA-DR allele polymorphism and multiple sclerosis in Chinese populations: a meta-analysis. *Multiple Sclerosis*.

[15] Rojas O. L., Rojas-Villarraga A., Cruz-Tapias P. (2010). HLA class II polymorphism in Latin American patients with multiple sclerosis. *Autoimmunity Reviews*.

[16] Zhang Q., Lin C. Y., Dong Q., Wang J., Wang W. (2011). Relationship between HLA-DRB1 polymorphism and susceptibility or resistance to multiple sclerosis in Caucasians: a meta-analysis of non-family-based studies. *Autoimmunity Reviews*.

[17] Mohajer B., Abbasi N., Pishgar F. (2018). HLA-DRB1 polymorphism and susceptibility to multiple sclerosis in the Middle East North Africa region: a systematic review and meta-analysis. *Journal of Neuroimmunology*.

[18] Rolim Lima T. F., Lopes Braga V. L., Diógenes Silva J. T. (2015). The HLA-DRB1 alleles effects on multiple sclerosis: a systematic review. *International Archives of Medicine*.

[19] Kaushansky N., Ben-Nun A. (2014). DQB1∗06:02-associated pathogenic anti-myelin autoimmunity in multiple sclerosis-like disease: potential function of DQB1∗06:02 as a disease-predisposing allele. *Frontiers in Oncology*.

[20] Verreck F. A., Termijtelen A., Koning F. (1993). HLA‐DR*β* chain residue 86 controls DR*αβ* dimer stability. *European Journal of Immunology*.

[21] Cocco E., Murru R., Costa G. (2013). Interaction between HLA-DRB1-DQB1 haplotypes in Sardinian multiple sclerosis population. *PLoS One*.

[22] Greer J. M., Pender M. P. (2005). The presence of glutamic acid at positions 71 or 74 in pocket 4 of the HLA-DR 1 chain is associated with the clinical course of multiple sclerosis. *Journal of Neurology, Neurosurgery, and Psychiatry*.

[23] Freed B. M., Schuyler R. P., Aubrey M. T. (2011). Association of the HLA–DRB1 epitope LA67, 74 with rheumatoid arthritis and citrullinated vimentin binding. *Arthritis and Rheumatism*.

