# Supplementary material for: The Involvement of HLA Class II Alleles in Multiple Sclerosis: A Systematic Review with Meta-analysis
Source: Dis Markers. 2019 Nov 6;2019:1409069. doi: 10.1155/2019/1409069 (PMC6875418; doi:10.1155/2019/1409069)
Supplement: Supplementary Materials — Supplementary data Figure 1: analysis of the quality of the studies included in the present review of systematic reviews. Supplementary data Figures 2-10: forest plots from the meta-analysis including HLA data from Rolim Lima et al. [file 1409069.f1.doc]

**
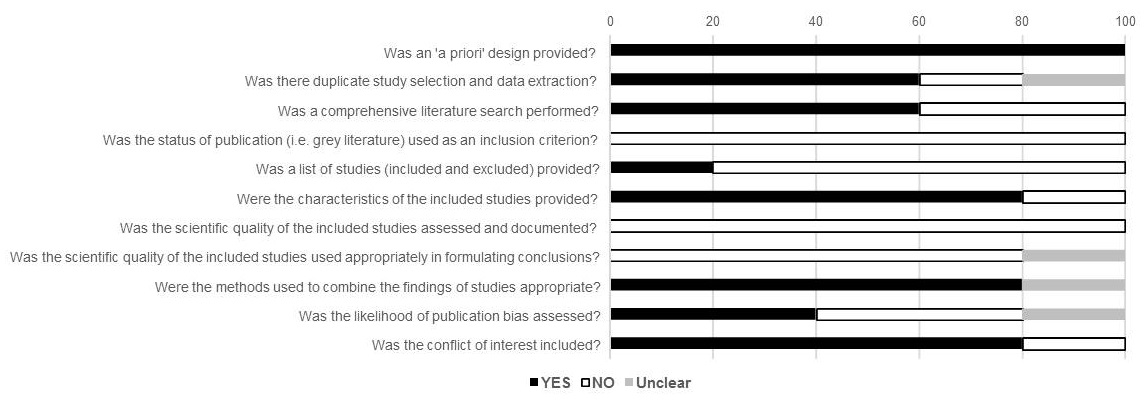
**

**Supplementary data Figure 1.** Analysis of the quality of the studies included in the present review of systematic reviews.


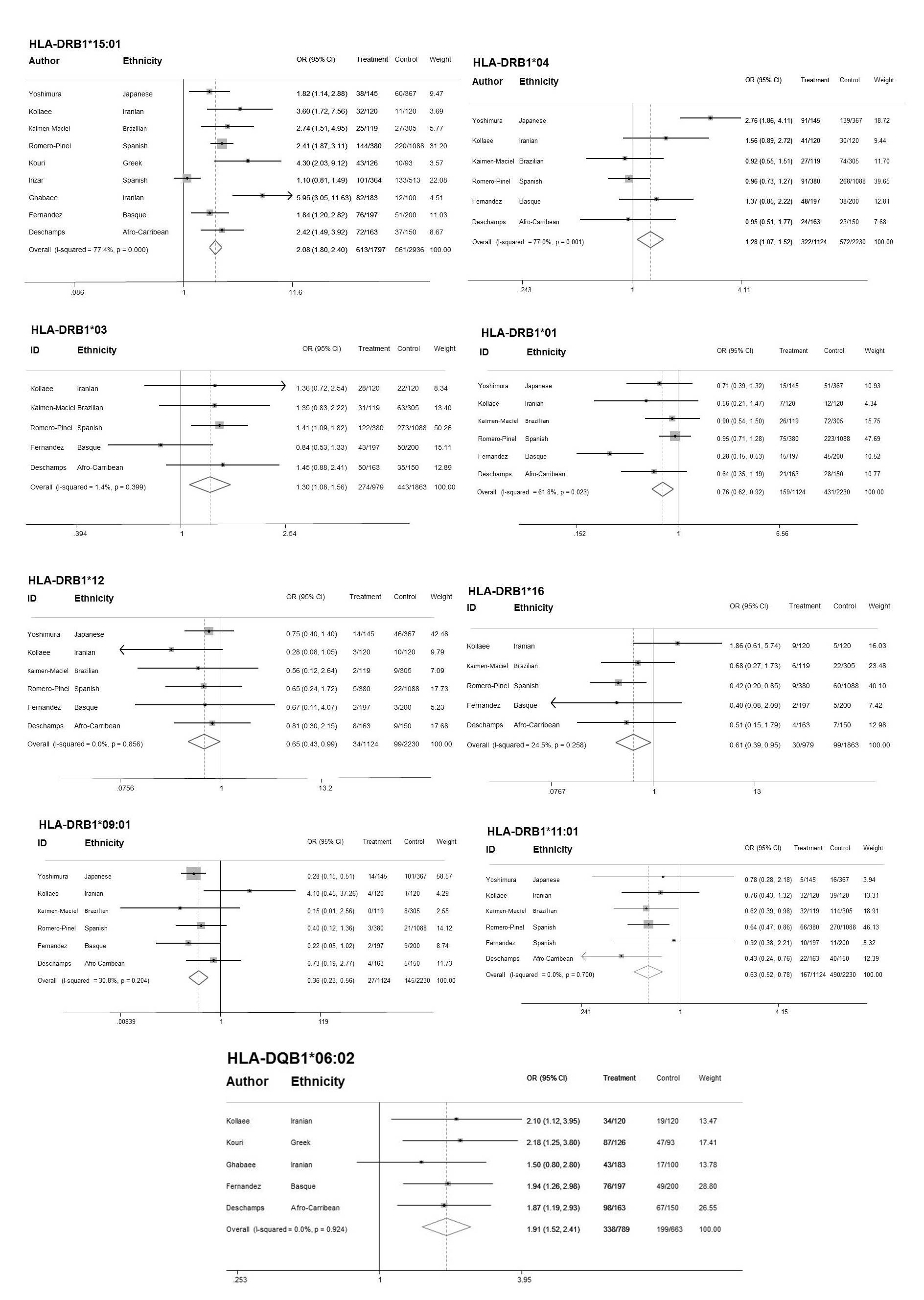


**Supplementary data Figure 2-10.** Forest plots from the meta-analysis including HLA data from Rolim Lima.
